# Konjunktur der Bundesländer: große Unterschiede, aber wenig aussagefähige Daten

**DOI:** 10.1007/s10273-021-2920-7

**Published:** 2021-05-17

**Authors:** Roland Döhrn

**Affiliations:** RWI - Leibniz-Institut für Wirtschaftsforschung e.V., Hohenzollernstr. 1-3, 45128 Essen, Deutschland

**Keywords:** C82, E01, R11

## Abstract

Im März 2021 legte der Arbeitskreis der Volkswirtschaftlichen Gesamtrechnungen der Länder die ersten Berechnungen zur Wirtschaftsleistung der Länder für 2020 vor. Es zeigen sich auch in der Corona-Krise deutliche Unterschiede zwischen den Ländern. Die Gründe hierfür sind vielfältig. Jedoch müssen diese vorläufigen Zahlen mit Vorsicht interpretiert werden, da die folgenden Revisionen die Positionen der Länder verändern können, wie der vorliegende Beitrag verdeutlicht.

## Abstract (en)

In March 2021, the first release of regional national accounts in 2020 was published. Due to the coronavirus crisis, GDP shrank in all federal states, but with significant differences. However, the data must be interpreted with caution. The regional national accounts are subject to large revisions, which may change the position of a federal state in a growth ranking considerably. In nine out of 17 years analysed, there is no significant correlation between the ranking of the states in the initially released data and in the final data. Thus, methods of regional national accounting should be improved to reduce revisions.

Der Arbeitskreis Volkswirtschaftliche Gesamtrechnungen der Länder (AK VGRdL) hat Ende März 2021 wieder erste Daten zur Entwicklung der Wirtschaftsleistung in den deutschen Ländern im Vorjahr vorgelegt. 2020 ist das reale Bruttoinlandsprodukt (BIP) in allen Ländern deutlich gesunken – angesichts der Corona-Krise keine Überraschung. Gleichwohl zeigen sich beachtliche Unterschiede: Während in Bremen (-7,0 %) und im Saarland (-6,7 %) das BIP deutlich stärker schrumpfte als in Deutschland insgesamt (-4,9 %), kamen Brandenburg und Mecklenburg-Vorpommern mit einem Rückgang von jeweils -3,2 % eher glimpflich davon.

Dies kann zahlreiche Gründe haben. So unterscheiden sich die Wirtschaftsstrukturen der Länder. Es zeigte sich auch diesmal, dass eher industriell geprägte Länder wie Bayern und Baden-Württemberg aufgrund ihrer intensiven Einbindung in die internationale Arbeitsteilung stärker unter globalen Konjunktureinbrüchen leiden als eher vom Dienstleistungssektor geprägte Länder wie Berlin. Auch waren die Länder unterschiedlich stark von der Pandemie betroffen und mussten mithin die wirtschaftliche Aktivität in unterschiedlichem Maße einschränken.

**Prof. Dr. Roland Döhrn** leitete bis 2020 den Kompetenzbereich „Wachstum, Konjunktur, Öffentliche Finanzen" am RWI – Leibniz-Institut für Wirtschaftsforschung in Essen.

Mit allzu weitreichenden Interpretationen der konjunkturellen Unterschiede 2020 zwischen den Ländern sollte man aufgrund der aktuellen Zahlen allerdings vorsichtig sein. Denn bei diesen handelt es sich um vorläufige Angaben, die erfahrungsgemäß noch in erheblichem Maße und weitaus stärker als die entsprechenden Angaben für Deutschland insgesamt revidiert werden. Im Zuge dieses Revisionsprozesses kann sich die Position der Länder im Wachstumsgefüge noch beträchtlich ändern.

## Grundlagen der Berechnung

Die VGRdL werden zunächst ausgehend von der Entstehungsseite erstellt.[1] Dabei verfolgt der Arbeitskreis einen – wie er es selbst bezeichnet – Pseudo-Bottom-Up-Ansatz. Tiefste Berechnungseinheit der Bruttowertschöpfung der Länder sind die Wirtschaftszweige. Da dazu aber auf regionaler Ebene vielfach statistische Informationen fehlen, wird die Bruttowertschöpfung häufig bestimmt, indem die Wertschöpfung in Deutschland insgesamt durch geeignete Schlüsselgrößen auf Länder heruntergebrochen wird.

Dabei durchläuft die VGRdL fünf Bearbeitungsrunden. Bei der ersten, deren Ergebnisse für 2020 nun veröffentlicht wurden, fehlen noch viele Ausgangsstatistiken, allen voran die Kostenstrukturerhebungen. Daher werden diese Daten vom Arbeitskreis auch als *Erste Fortschreibung* bezeichnet. Ein Jahr später wird die zweite Fortschreibung erstellt, in die neben anderen in der Zwischenzeit veröffentlichten

1 Angaben zur Verwendungs- und Verteilungsseite des BIP werden erst mit größerem zeitlichem Abstand erstellt. Eine ausführliche Darstellung der Berechnungsmethoden gibt AK VGRdL (2017).

Tabelle 1

**Revision der Veränderungsrate des realen BIP in den Ländern**

Mittlere absolute Revision

| | Kumuliert gegen 1. Fortschreibung | | | | Gegenüber jeweils voriger Revision | | | Nachrichtlich: Wurzel aus der mittleren quadratischen Revision[1] |
|---|---|---|---|---|---|---|---|---|
| | 2. FS 2000 – 19 (1) | 1. OB 2000 – 18 (2) | 2. OB 2000 – 17 (3) | 3. OB 2000 – 16 (4) | 1. OB 2000 – 18 (5) | 2. OB 2000 – 17 (6) | 3. OB 2000 – 16 (7) | (8) |
| Baden-Württemberg | 0,46 | 0,92 | 0,88 | 0,94 | 0,93 | 0,06 | 0,07 | 1,13 |
| Bayern | 0,35 | 0,64 | 0,68 | 0,78 | 0,40 | 0,12 | 0,14 | 1,06 |
| Berlin | 0,38 | 0,90 | 1,16 | 1,21 | 1,00 | 0,33 | 0,07 | 1,42 |
| Brandenburg | 0,40 | 1,14 | 0,98 | 1,13 | 1,90 | 0,22 | 0,40 | 1,49 |
| Bremen | 0,80 | 1,26 | 1,19 | 1,31 | 1,72 | 0,42 | 0,21 | 1,73 |
| Hamburg | 0,46 | 1,16 | 1,21 | 1,32 | 1,92 | 1,16 | 1,12 | 1,62 |
| Hessen | 0,45 | 0,82 | 0,80 | 0,80 | 0,63 | 0,19 | 0,10 | 1,05 |
| Mecklenburg-Vorpommern | 0,50 | 0,88 | 0,81 | 0,82 | 0,95 | 0,28 | 0,59 | 1,02 |
| Niedersachsen | 0,75 | 0,92 | 1,03 | 1,04 | 0,50 | 0,13 | 0,15 | 1,51 |
| Nordrhein-Westfalen | 0,26 | 0,57 | 0,54 | 0,60 | 0,38 | 0,12 | 0,08 | 0,76 |
| Rheinland-Pfalz | 0,55 | 0,67 | 0,69 | 0,59 | 0,54 | 0,10 | 0,10 | 0,74 |
| Saarland | 0,60 | 1,02 | 1,07 | 1,08 | 2,12 | 0,17 | 0,17 | 1,30 |
| Sachsen | 0,33 | 0,56 | 0,75 | 0,76 | 0,43 | 0,18 | 0,19 | 0,95 |
| Sachsen-Anhalt | 0,56 | 1,01 | 0,95 | 1,05 | 1,24 | 0,12 | 0,16 | 1,36 |
| Schleswig-Holstein | 0,47 | 0,83 | 0,97 | 0,98 | 0,50 | 0,11 | 0,14 | 1,21 |
| Thüringen | 0,41 | 0,67 | 0,71 | 0,92 | 1,15 | 0,07 | 0,16 | 1,12 |
| Bund | 0,07 | 0,20 | 0,26 | 0,34 | 0,06 | 0,02 | 0,04 | 0,42 |

Anmerkungen: FS: Fortschreibung. OB: Originärberechnung. [1] Dritte Originärberechnung gegenüber erster Fortschreibung, 2000 bis 2016.

Quelle: eigene Berechnungen nach Angaben des AK VGRdL.

Ausgangsstatistiken auch eine provisorische Kostenstrukturerhebung für das Verarbeitende Gewerbe eingearbeitet ist. Der volle Satz an Kostenstrukturerhebungen liegt allerdings erst im darauffolgenden Jahr vor, sodass erst die dritte Überarbeitung als *Originärberechnung* bezeichnet wird. Diese wird für 2020 voraussichtlich im März 2023 publiziert. In den nachfolgenden beiden Bearbeitungsrunden werden weitere Ausgangsstatistiken eingearbeitet und die Ergebnisse an die Revisionen der gesamtdeutschen VGR angepasst, sodass die *endgültigen* VGRdL erst nach fünf Jahren vorliegen – für 2020 also erst im März 2025.[2]

## Revisionen führen zu Verschiebungen im Wachstums-Ranking

Tabelle 1 zeigt das Ausmaß der Revisionen der Veränderungsraten des preisbereinigten BIP im Zuge dieser Überarbeitungsrunden.[3] Sie verdeutlicht *erstens*, dass die Revisionen bei allen Ländern auf allen Stufen des Revisionsprozesses beträchtlich größer ausfallen als die für das deutsche BIP insgesamt. *Zweitens* sind die Revisionen auf dem Weg zum endgültigen Wert bei großen Ländern in der Summe tendenziell kleiner als bei kleinen (Spalte 4). Darin dürfte zum Ausdruck kommen, dass die Entwicklung letzterer stärker von einzelnen Branchen, oft sogar Unternehmen geprägt ist. Der Zusammenhang ist aber nicht strikt; auch die Revisionen des BIP beispielsweise von Rheinland-Pfalz und Sachsen sind relativ gering. *Drittens* zeigt die Tabelle, dass in den meisten Ländern die größten Revisionen beim Übergang von der zweiten Fortschreibung zur ersten Originärberechnung erfolgen (Spal-

2 Danach kann es im Zuge von Generalrevisionen der VGR zu weiteren Änderungen der Angaben der VGRdL kommen, auf die hier nicht weiter eingegangen wird.

3 Dabei kann nicht zwischen sogenannten laufenden Revisionen und Generalrevisionen unterschieden werden. Nach Angaben des AK VGRdL betrug z. B. die absolute Revision der BIP-Raten im Durchschnitt aller Länder aufgrund der Generalrevision 2019 0,25 Prozentpunkte von 1999 bis 2018. Im Vergleich dazu betrug die analog berechnete mittlere absolute Revision des finalen Jahresergebnisses gegenüber der ersten Veröffentlichung 0,98 Prozentpunkte (AK VGRdL, 2020, 15).

Abbildung 1
**68 %-Revisionsbänder der Zunahme des realen BIP, 2020**

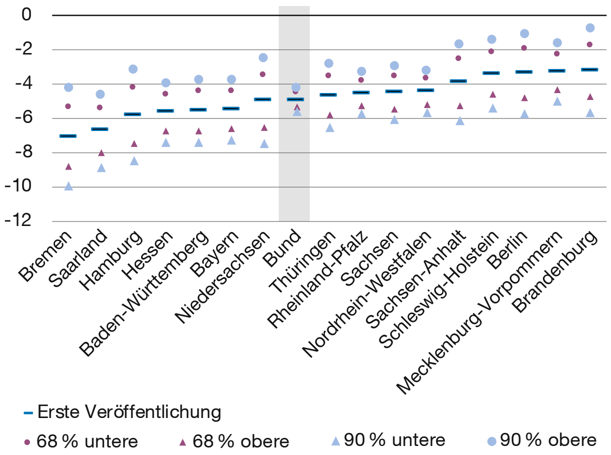

Quelle: eigene Berechnungen.

Abbildung 2
**Rangstabilität der Länder hinsichtlich der Zunahme des realen BIP**
Rangkorrelationen

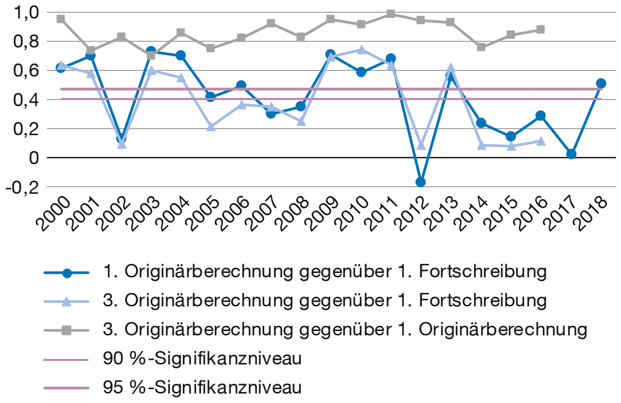

Quelle: eigene Berechnungen.

te 5). Dies war zu erwarten, weil sich in diesem Rechenschritt die Verfügbarkeit an Primärstatistiken am stärksten verbessert. *Viertens* fällt auf, dass das Ausmaß der Revisionen gegenüber der Erstveröffentlichung im Zuge des Revisionsprozesses nicht kontinuierlich zunimmt, sondern auch wieder zurückgehen kann, dass also der im Laufe der Zeit veröffentlichte Wert um den endgültigen schwanken kann. *Schließlich* ist die mittlere quadratische Revision (Spalte 8) in der Regel nur geringfügig größer als die zeitlich vergleichbare mittlere absolute (Spalte 4). Dies weist darauf hin, dass die Revisionen sich relativ gleichmäßig auf die Jahre des Beobachtungszeitraums verteilen, die Ergebnisse also nicht durch ungewöhnlich große Revisionen in einzelnen Jahren geprägt sind.

Da die Revisionen in der Regel unverzerrt sind (Döhrn 2021a), kann man die Wurzel aus der mittleren quadratischen Revision als deren Standardfehler interpretieren. Diesen kann man wiederum verwenden, um Konfidenzbänder der jüngst veröffentlichten Daten für 2020 zu berechnen.[4] Abbildung 1 zeigt (Normalverteilung der Revisionen unterstellt) das 68 %- und das 90 %-Intervall. Die Bänder sind in allen Ländern sehr weit, beträchtlich weiter als beim Bund. Wichtiger noch: Die Konfidenzbänder der Länder überlappen sich erheblich. Darin deutet sich bereits an, dass sich die Rangfolge der Länder hinsichtlich der Höhe der Expansion des BIP im Zuge des Revisionsprozesses erheblich verändern kann. Dies war auch in der Vergangenheit in den meisten Jahren der Fall, wie Abbildung 2 zeigt: Lediglich in acht und damit in weniger als der Hälfte der 17 betrachteten Jahre bestand eine signifikante Korrelation zwischen der Rangfolge der Länder hinsichtlich der BIP-Raten bei der ersten Fortschreibung der VGRdL und der Rangfolge nach Abschluss des Revisionsprozesses.

Aus alledem kann man zunächst zwei Schlussfolgerungen ziehen: *Erstens* muss man die aktuellen Daten der VGRdL mit Vorsicht genießen und keine voreiligen Schlüsse ziehen, also auch nicht dazu, welches Land die Corona-Pandemie besser bewältigt hat als andere. *Zweitens* sind die großen Revisionsintervalle ärgerlich für die Öffentlichkeit, da sie erst mit großem zeitlichem Abstand erfährt, wie die wirtschaftliche Entwicklung des Landes war.[5] Daher sollte nach Möglichkeit versucht werden, die Revisionsanfälligkeit der VGRdL zu verringern. Ein Ansatzpunkt wäre, Arbeitsmarktkennziffern ein höheres Gewicht bei der Berechnung einzuräumen, da sie zum einen trennschärfer Regionen zugeordnet werden können als kapitalbezogene Wertschöpfungskomponenten, und diese zum anderen auch früher vorliegen und selbst weniger revisionsanfällig sind. Ein solches Vorgehen könnte allerdings zu von der bisherigen Praxis abweichenden Angaben zur Wertschöpfung auf Länderebene führen.

4 Dies impliziert allerdings, dass die in der Vergangenheit beobachteten Revisionen sich auf die aktuelle, sicherlich durch besondere Bedingungen gekennzeichnete Situation übertragen lassen.

5 Erinnert sei an die 2016 in Nordrhein-Westfalen geführte „Nullwachstumsdebatte“ nachdem für 2015 eine BIP-Rate von 0 % veröffentlicht worden war. Inzwischen steht eine Rate von 1,6 % zu Buch, nur knapp unter dem Bundesdurchschnitt.

Abbildung 3

**Zuwachsrate des realen BIP und Beurteilung der Wirtschaftslage in den Ländern, 2016**

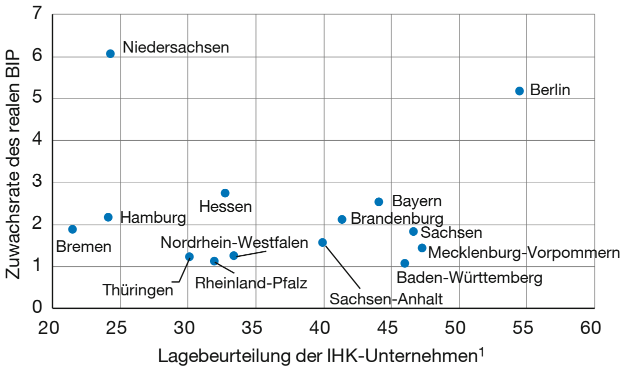

[1] Saldenindex berechnet als Differenz der Anteile der Unternehmen mit der Lagebeurteilung gut und schlecht. Mittelwert aus drei bzw. vier jährlichen Befragungen.

Quelle: eigene Berechnungen.

## Unterschiede in den BIP-Raten spiegeln sich nicht im Stimmungsbild der Wirtschaft wider

Dies eröffnet eine Diskussion darüber, ob wir das BIP auf Länderebene überhaupt richtig messen. Generell gehen in den Volkswirtschaftlichen Gesamtrechnungen Definition und Berechnung Hand in Hand. Die Statistischen Landesämter definieren also, was als regionales BIP gilt. Allerdings sollten die Ergebnisse ihrer Rechnungen auch mit Informationen aus anderen Quellen harmonieren, vorzugsweise mit solchen Informationen, die weder in die Berechnung der VGRdL eingehen noch durch die Ergebnisse der VGRdL beeinflusst werden.

Eine solche Information sind beispielsweise die Konjunkturumfragen der Industrie- und Handelskammern (IHK). Diese werden in allen Kammerbezirken nach einer vergleichbaren, wenn auch nicht identischen Methodik dreibzw. viermal im Jahr durchgeführt. Die Ergebnisse auf IHK-Ebene werden auch zu Landesergebnissen zusammengefasst.[6] Im Zeitablauf korreliert die Einschätzung der Wirtschaftslage der IHK-Unternehmen recht eng mit dem Zuwachs des BIP der Länder. Naheliegend wäre, dass dies auch im Querschnitt gilt, dass also dort, wo sich die Wirtschaft im Vergleich der Länder laut VGRdL günstiger entwickelte, auch die IHK befragten Unternehmen ihre Lage überdurchschnittlich gut einschätzen und umgekehrt.

Abbildung 3 zeigt den Zusammenhang zwischen BIP-Rate und dem IHK-Lageindex 2016, also dem derzeit letzten Jahr, für das endgültige Werte der VGRdL vorliegen. Es verdeutlicht, dass zwischen beiden Größen kein Zusammenhang besteht. Dies ist zwar nur eine Momentaufnahme, aber in anderen Jahren sieht es nicht viel besser aus (Döhrn, 2021b, 20). Der fehlende Zusammenhang zwischen beiden Größen ist zwar noch kein Beweis, dass die VGRdL ein nicht zutreffendes Bild der konjunkturellen Lage in den Ländern zeichnen. So deckt die IHK-Befragung nicht das gesamte Spektrum der Wirtschaftsbereiche ab.[7] Allerdings könnte das Ergebnis zum Anlass genommen werden, die Ergebnisse der VGRdL kritisch zu hinterfragen.

6 Vgl. zur Methodik der Konjunkturumfragen der IHK und zur Berechnung der Jahreswerte Döhrn (2021b, 5-10).

7 Da der staatliche Bereich, die Landwirtschaft und zumeist auch das Handwerk nicht den IHK angehören, fehlen sie in der Befragung. Aber auch in Bereichen wie dem Verarbeitenden Gewerbe, wo eine hohe Abdeckung der wirtschaftlichen Aktivität durch die IHK-Befragung gegeben ist, findet man in den meisten Jahren keinen Zusammenhang zwischen Lagebeurteilung und Zunahme der realen Bruttowertschöpfung (Döhrn, 2021b).
